# Ultraviolet Photo-Activated NO_2_ Gas Sensors Based on P-Type Gold-Black Nanoparticles/N-Type Ga_2_O_3_ Nanorods Grown on Nanoimprinted SnO_2_ Seed Layer

**DOI:** 10.3390/mi17101208

**Published:** 2026-10-21

**Authors:** Tun-Hui Yang, Mu-Ju Wu, Wen-Chieh Lee, Ching-Ting Lee, Chun-Hung Lin, Hsin-Ying Lee

**Affiliations:** 1 Department of Photonics, National Cheng Kung University, Tainan 701, Taiwan; cherry.tseng40214@gmail.com (T.-H.Y.); jj.s.lee0314@gmail.com (W.-C.L.); ctlee@ee.ncku.edu.tw (C.-T.L.); chlin@ncku.edu.tw (C.-H.L.); 2 Program on Key Materials, Academy of Innovative Semiconductor and Sustainable Manufacturing, National Cheng Kung University, Tainan 701, Taiwan; m20001115@gmail.com; 3 Institute of Microelectronics, Department of Electrical Engineering, National Cheng Kung University, Tainan 701, Taiwan; 4 Department of Electrical Engineering, Yuan Ze University, Taoyuan 320, Taiwan

**Keywords:** Ga_2_O_3_ nanorods, gold-black nanoparticles, nanoimprint system, NO_2_ gas sensor, ultraviolet light illumination

## Abstract

This study aimed to enhance the sensing performance of nitrogen dioxide (NO_2_) gas sensors fabricated using gallium oxide (Ga_2_O_3_) nanorod sensing membranes. The hydrothermal synthesis method was used to grow GaOOH nanorods on a roughened tin oxide (SnO_2_) seed layer with various periods fabricated using a nanoimprint system. Then, the GaOOH nanorods were converted to Ga_2_O_3_ nanorods by nitrogen annealing treatment. Under 10 ppm NO_2_ and operating temperature of 210 °C, the optimal response of the NO_2_ gas sensors using Ga_2_O_3_ nanorods grown on an 800 nm periodic seed layer was 56.2. To further improve the performance attributed to the spillover effect of noble metal, p-type gold-black nanoparticles (NPs) were deposited onto the n-type Ga_2_O_3_ nanorod surfaces using a vapor cooling condensation system to form p-n heterojunctions. When Ga_2_O_3_ nanorods were decorated with 1.02 at.% gold content, the optimal response was increased to 143.9 under 10 ppm NO_2_ at an operating temperature of 180 °C. To investigate the photo-activation effect on the NO_2_ gas sensors, 245 nm UV light was applied to the devices. Under the UV light intensity of 4.8 mW/cm^2^, the response of the resulting NO_2_ gas sensors without and with gold-black NPs was increased to 123.1 and 370.5, respectively.

## 1. Introduction

In recent years, environmental pollution has become increasingly severe due to the rapid development of industrial activities. Among various air pollutants, nitrogen dioxide (NO_2_) in the atmosphere has raised significant concern, as it can cause respiratory tract irritation, pulmonary diseases, and even lead to death in severe cases [1]. Therefore, the development of NO_2_ gas sensors with high response and fast response has become essential to address the associated health and environmental risks.

Over the past few decades, metal oxide semiconductor (MOS) gas sensors have garnered extensive attention due to their advantages, including simple fabrication processes, low cost, ease of operation, and potential for miniaturization. As a result, numerous metal oxide semiconductors have been investigated as sensing membranes for NO_2_ detection. These include a variety of n-type and p-type semiconductors such as zinc oxide (ZnO) [2], titanium dioxide (TiO_2_) [3], tin oxide (SnO_2_) [4], tungsten trioxide (WO_3_) [5], indium oxide (In_2_O_3_) [6], gallium oxide (Ga_2_O_3_) [7], nickel oxide (NiO) [8], etc. Among the above-mentioned materials, Ga_2_O_3_ is a promising material for gas-sensing applications because of its wide bandgap, high thermal stability, and chemical robustness, making it particularly suitable for high-temperature and harsh-environment sensing. Consequently, many Ga_2_O_3_-based gas sensors have been developed for operation at relatively high temperatures [9,10,11]. In addition, nanostructured materials have attracted increasing interest in the field of gas sensing owing to their high surface-to-volume ratio, which provides more active adsorption sites for sensing gas molecules [12]. Compared to conventional thin-film gas sensors, nanostructures offer significantly enhanced sensitivity and faster response and recovery times, thereby improving overall gas sensor performance. Various morphologies have been studied, including nanorods [13], nanosheets [14], nanospheres [15], nanotubes [16], and nanowires [17]. To enhance the response of gas sensors, various strategies have been explored, including the modification of nanostructures [18], construction of heterojunctions [19], incorporation of composite materials [20], decoration with noble metals [21], and elemental doping [22]. Among these strategies, the surface decoration of nanomaterials with noble metals, such as gold (Au), silver (Ag), platinum (Pt), and palladium (Pd), has been proven to be particularly effective in enhancing chemical sensitization. Noble metals act as catalysts, facilitating the dissociation and adsorption of oxygen molecules on the sensing material surface and inducing the spillover effect, thereby expanding the reactive area and improving charge-transfer efficiency. These effects collectively result in significantly improved sensor response and reaction kinetics [23,24]. In addition to noble metal decoration, ultraviolet (UV) light activation is another effective approach for improving sensing performance. UV light illumination generates photo-generated electron-hole pairs, thereby enhancing the participation of charge carriers in surface reactions and increasing sensitivity [25,26]. These findings confirm that noble metal decoration and UV light illumination are highly promising strategies for significantly improving the sensitivity, response rate, and operational stability of metal oxide gas sensors, thereby paving the way for their application in practical, low-power, and high-performance sensing platforms.

In this work, to enhance the response of NO_2_ gas sensors, the hydrothermal synthesis method was utilized to grow GaOOH nanorods on SnO_2_ seed layers with various periodic parallel-strip patterns formed by using a nanoimprint system. The GaOOH nanorods were converted to the n-type Ga_2_O_3_ nanorods via annealing. To investigate the response enhancement mechanisms of the p-n heterojunction and the spillover effect, various contents of p-type gold-black nanoparticles (NPs) were deposited onto the surface of the Ga_2_O_3_ nanorods using a vapor cooling condensation system. The carrier type of gold-black NPs was verified as p-type in the literature [27]. The morphology of the resulting Ga_2_O_3_ nanorods was carried out using a scanning electron microscope (SEM, AURIGA, ZEISS, Oberkochen, Germany). The morphology of the gold-black NPs/Ga_2_O_3_ nanorods was observed using a high-resolution transmission electron microscope (HRTEM, JEM-2100F, JEOL Ltd., Tokyo, Japan). Furthermore, to study the light-activated effect, UV light was illuminated onto various NO_2_ gas sensors. The current–time (I–t) characteristics of various NO_2_ gas sensors were measured using an Agilent 4156C semiconductor parameter analyzer (Keysight Technologies, Santa Rosa, CA, USA). To evaluate sensing performance, the characteristics of the NO_2_ gas sensors were measured across various temperatures, NO_2_ concentrations, and relative humidity (RH) levels.

## 2. Materials and Methods

After the 20 μL poly(methyl methacrylate) (PMMA, CHIMEI Corporation Co., Ltd., Tainan, Taiwan) solution (5 wt.% PMMA in 95 wt.% toluene, Sigma-Aldrich Co. (Taiwan Merck), Taipei City, Taiwan) was spin-coated on quartz substrates with a rotating speed of 3000 rpm for 30 s, the substrates were baked on a hot plate at 150 °C for 20 min to remove the organic solvent. Subsequently, the perfluoropolyether (PFPE, Syensqo SA/NV, Brussels, Belgium) working molds with different periods of 600 nm, 800 nm, and 1000 nm were placed onto the PMMA-coated substrates. Then the entire set was imprinted at 150 °C under a pressure of 3 bar for 20 min in a nanoimprint system to form the PMMA parallel-strip array structures with different periods. Details of the PFPE mold nanoimprinting process could be found in the literature [28,29]. Finally, the residual PMMA layer was removed using an oxygen (O_2_) plasma etching system with an RF power of 30 W, an O_2_ gas flow of 90 sccm, a chamber pressure of 50 mtorr, and an etching time of 25 s. Consequently, the roughened PMMA morphologies were formed. A 50 nm thick SnO_2_ layer was then deposited on various periodic PMMA strip array structures using a radio frequency (RF) magnetron sputtering system, forming roughened SnO_2_ seed layers with different periods. During the SnO_2_ deposition procedure, the RF power, Ar/O_2_ gas flow ratio, and the chamber pressure were 75 W, 48/2 sccm, and 5 mtorr, respectively. The GaOOH nanorods were subsequently grown on the roughened SnO_2_ seed layer using the hydrothermal synthesis method with the precursor of gallium nitrate hydrate (Ga(NO_3_)_3_·9H_2_O, Sigma-Aldrich Co. (Taiwan Merck), Taiwan) (12 mM) and hexamethylenetetramine (C_6_H_12_N_4_, HMT, Sigma-Aldrich Co. (Taiwan Merck), Taiwan) (10 mM) at 180 °C for 4 h. The grown GaOOH nanorods were then converted to Ga_2_O_3_ nanorods by annealing in a highly pure nitrogen (N_2_) atmosphere at 400 °C for 2 h. After annealing treatment at 400 °C, the PMMA was volatilized. The hydrothermal synthesis chemical formulas were expressed as [30]:

C_6_H_12_N_4_ + 6H_2_O → 6HCHO + 4NH_3_ (1)

NH_3_ + H_2_O → NH_4_^+^ + OH^–^ (2)

Ga^3+^ + 3OH^–^ → GaOOH + H_2_O (3)

2GaOOH → Ga_2_O_3_ + H_2_O (4)

**Figure 1:**
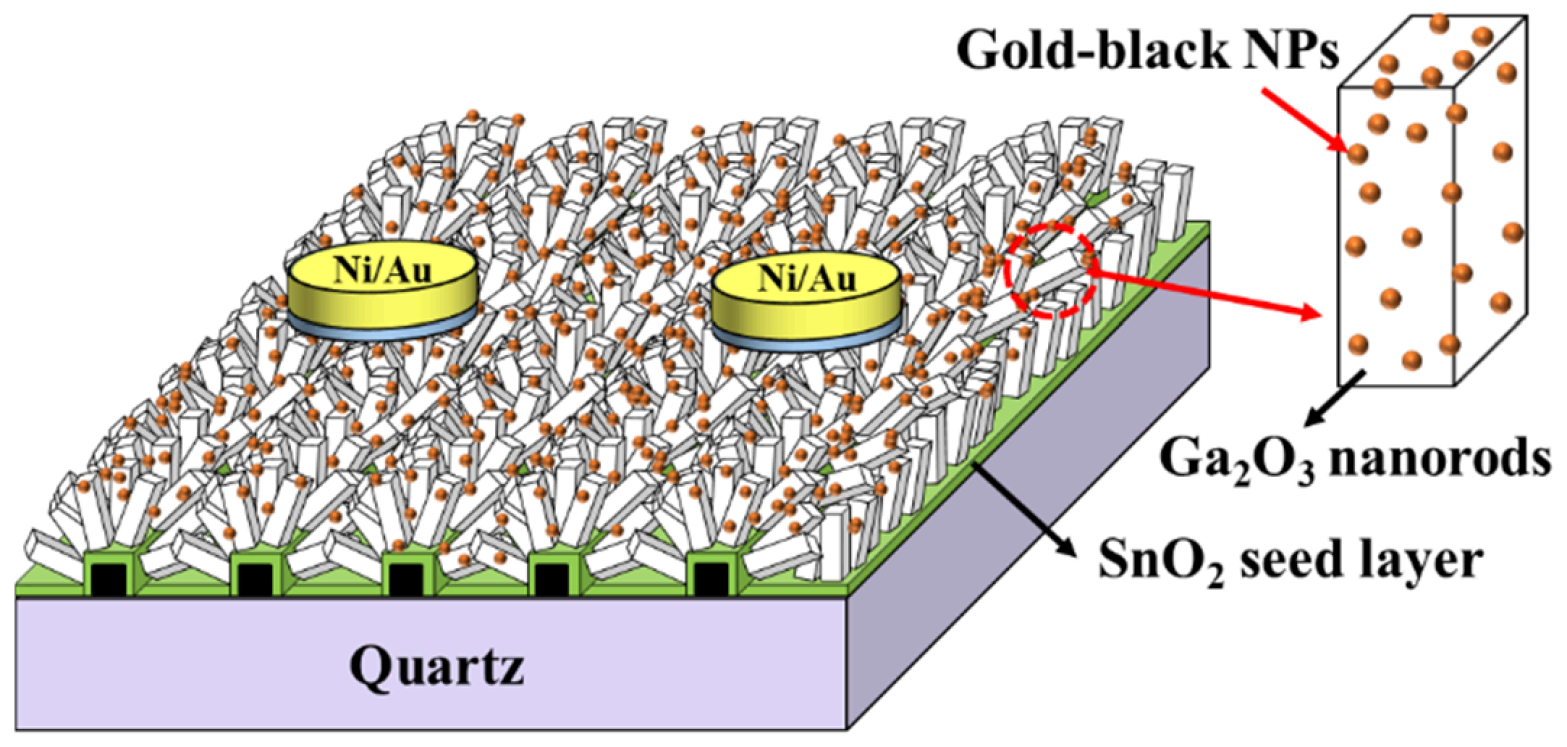
Schematic configuration of NO_2_ gas sensor fabricated using sensing membranes composed of gold-black NP-decorated Ga_2_O_3_ nanorods grown on roughened SnO_2_ seed layer.

To form p-n heterojunctions, the p-type gold-black NPs were deposited on the n-type Ga_2_O_3_ nanorods using a vapor cooling condensation system for 30, 60, and 90 s, respectively. When the chamber pressure of the vapor cooling condensation system was reduced to 300 mtorr, the tungsten boat was heated to sublimate Au, allowing Au vapor to condense onto the surface of the Ga_2_O_3_ nanorods, with the sample attached to the liquid nitrogen-cooled holder. Subsequently, nickel/gold (Ni/Au) metals (25/100 nm) were deposited as electrodes for the NO_2_ gas sensors using an electron-beam evaporator. Figure 1 shows the schematic configuration of the resulting NO_2_ gas sensors fabricated using sensing membranes composed of gold-black NP-decorated Ga_2_O_3_ nanorods grown on a roughened SnO_2_ seed layer. The sensing area of the NO_2_ gas sensors was 5 × 5 mm^2^.

Upon exposure to NO_2_ gas, NO_2_ molecules interacted with the Ga_2_O_3_ nanorod sensing membrane, resulting in an increase in the resistance of the NO_2_ sensor. This change in resistance was primarily attributed to the electron-withdrawing nature of NO_2_, which captured electrons from the conduction band of the Ga_2_O_3_ nanorods. The surface reaction mechanisms were expressed as [31]:

O_2(gas)_ → O_2(ads)_ (5)

(6) $$O_{2(\mathrm{ads})}+e^{-}\to \;O_{2(\mathrm{ads})}^{-}\;\;<100\;\mathrm{{}^{\circ}C}$$

(7) $$O_{2(\mathrm{ads})}^{-}+e^{-}\to \;{2O}_{\left(\mathrm{ads}\right)}^{-}\;\;100\;\mathrm{{}^{\circ}C}\sim 300\;\mathrm{{}^{\circ}C}$$

(8) $${\mathrm{NO}}_{2(\mathrm{gas})}+e^{-}\to {\mathrm{NO}}_{2(\mathrm{ads})}^{-}$$

(9) $${\mathrm{NO}}_{2(\mathrm{gas})}+O_{\left(\mathrm{ads}\right)}^{-}\to {\mathrm{NO}}_{2(\mathrm{ads})}^{-}+1/2O_{2(\mathrm{gas})}$$

The variation in resistance served as an indicator for evaluating the response of the NO_2_ gas sensors. Once the test chamber was purged with air to remove the residual NO_2_ molecules, the previously trapped electrons were thermally released, allowing the sensor resistance to recover to its initial baseline value.

## 3. Results and Discussion

**Figure 2:**
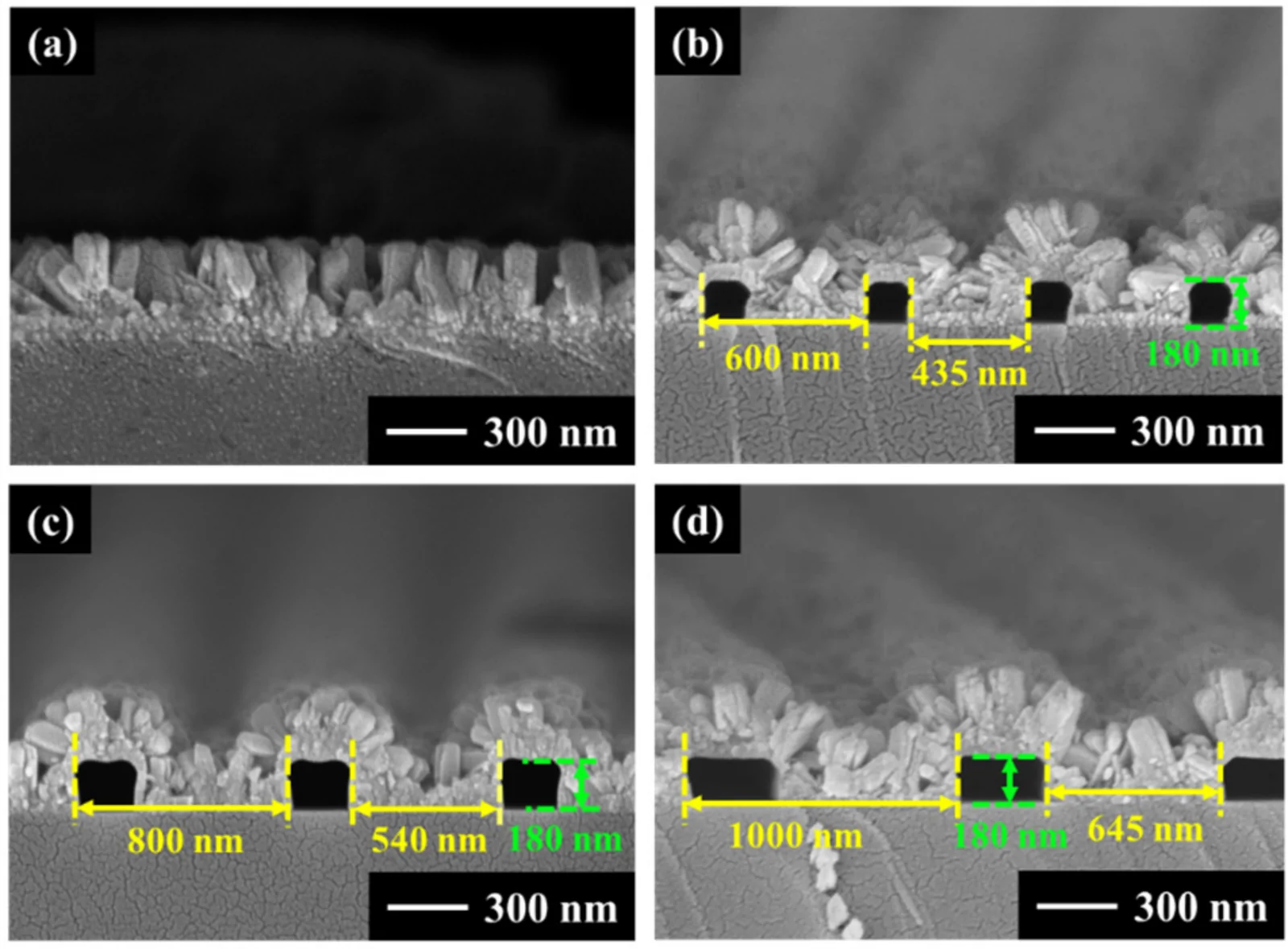
SEM cross-sectional images of Ga_2_O_3_ nanorods grown on (**a**) planar SnO_2_ seed layer, and roughened SnO_2_ seed layer with period of (**b**) 600 nm, (**c**) 800 nm, and (**d**) 1000 nm.

**Figure 3:**
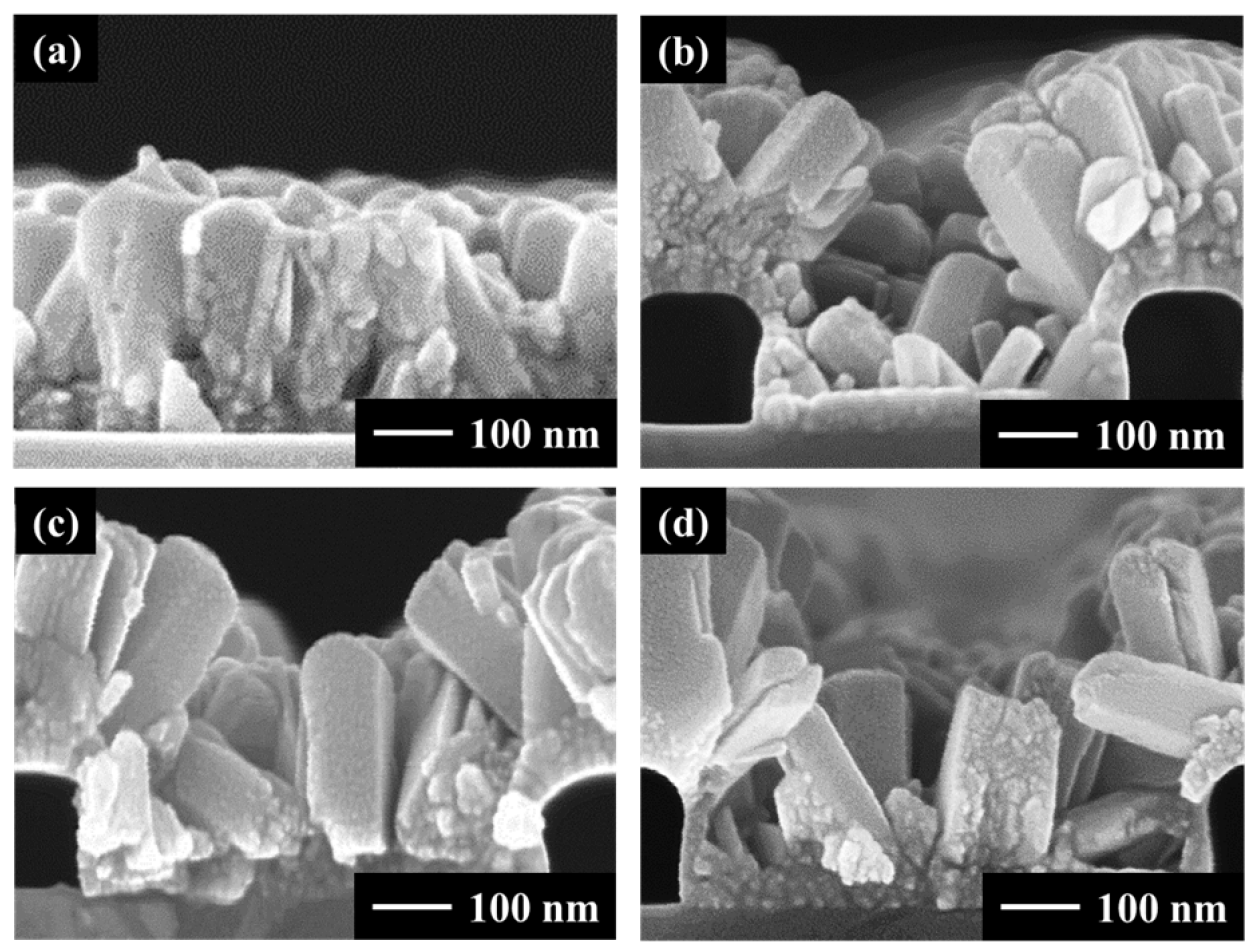
Enlarged SEM cross-sectional images of Ga_2_O_3_ nanorods grown on (**a**) planar SnO_2_ seed layer, and roughened SnO_2_ seed layer with period of (**b**) 600 nm, (**c**) 800 nm, and (**d**) 1000 nm.

Figure 2a–d show the SEM cross-sectional images of the Ga_2_O_3_ nanorods grown on the planar SnO_2_ seed layer and roughened SnO_2_ seed layers with a period of 600 nm, 800 nm, and 1000 nm, respectively. It could be observed that the widths of the groove regions of the periodic structures with a period of 600 nm, 800 nm, and 1000 nm were 435 nm, 540 nm, and 645 nm, respectively. The corresponding widths of the protruding regions were 165 nm, 260 nm, and 355 nm, respectively. The height of the protruding regions for the three kinds of roughened SnO_2_ seed layers was 180 nm. It was to be expected that because more sidewalls were obtained from the periodic groove regions, the roughened SnO_2_ seed layers with various periods had a larger surface area compared with the planar seed layer. According to the SEM measurement results, under the same patterned area of 5 mm × 5 mm, the estimated surface area increases relative to the planar seed layer were approximately 60%, 45%, and 36% for the 600, 800, and 1000 nm periodic structures, respectively. Figure 3a–d show the enlarged SEM cross-sectional images of Ga_2_O_3_ nanorods corresponding to Figure 2a–d, respectively. As shown in Figure 3a, most Ga_2_O_3_ nanorods growing on the planar SnO_2_ seed layer grew vertically upward. However, as shown in Figure 3b–d, in addition to the vertically grown Ga_2_O_3_ nanorods, Ga_2_O_3_ nanorods that grew on the sidewalls of the protruding areas grew laterally, thereby increasing the total number of Ga_2_O_3_ nanorods. As shown in Figure 3b, the shorter Ga_2_O_3_ nanorods were grown on the groove regions of the 600 nm periodic SnO_2_ seed layer due to the suppression by the narrower groove regions. In contrast, as shown in Figure 3c,d, because there was enough area in the groove region to grow Ga_2_O_3_ nanorods, it was possible to grow tilted Ga_2_O_3_ nanorods of similar length of 230 nm and diameter of 100 nm to the vertical Ga_2_O_3_ nanorods. However, in a unit area, since the 800 nm periodic protruding regions had more sidewall region than the 1000 nm periodic protruding regions, a greater total number of Ga_2_O_3_ nanorods could be grown.

**Figure 4:**
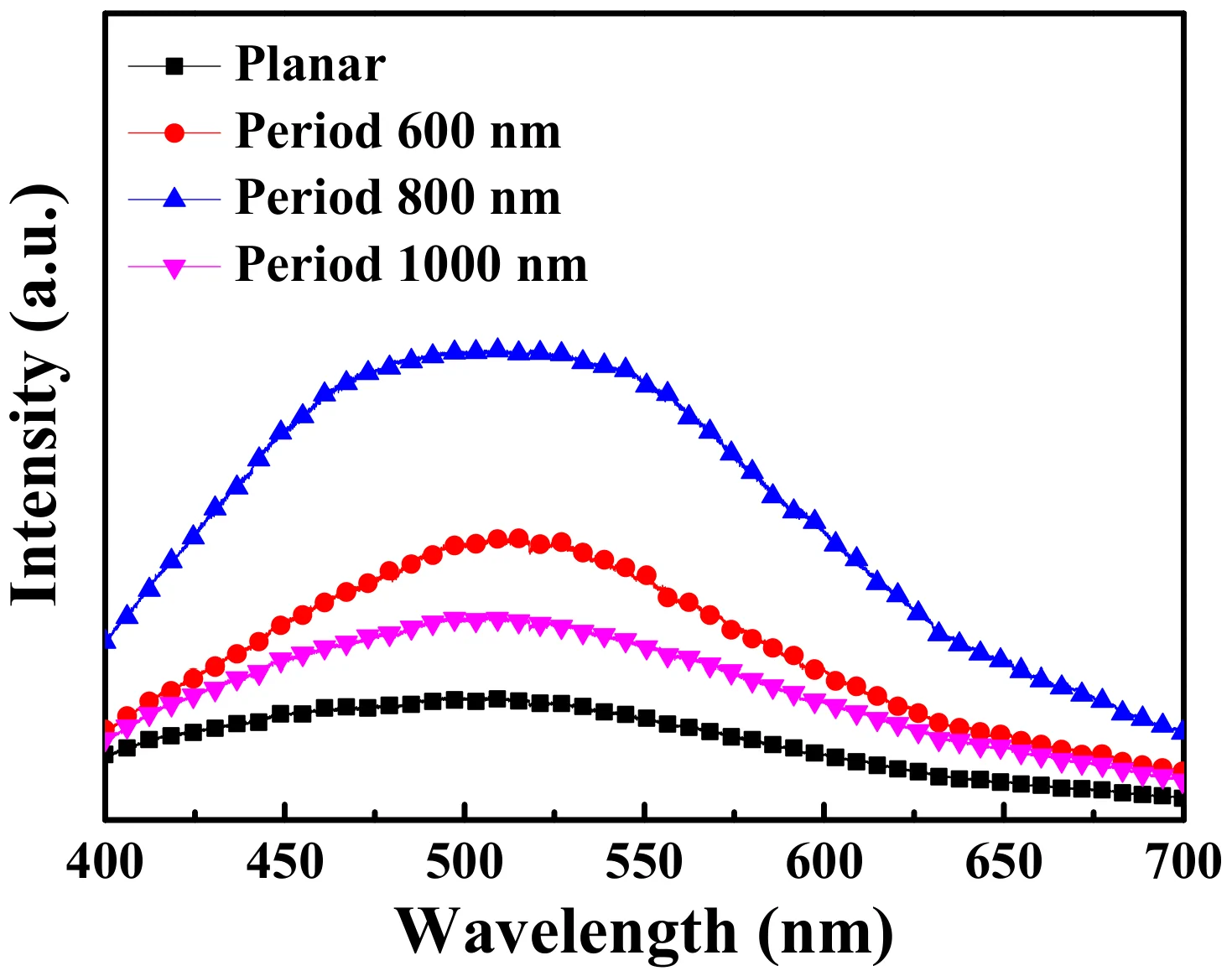
Photoluminescence spectra of Ga_2_O_3_ nanorods grown on planar and various periodic-roughened SnO_2_ seed layers.

In gas sensors, the gas adsorption site plays an important role. The gas adsorption sites could be associated with oxygen vacancy-related defects on the Ga_2_O_3_ nanorods. In this study, room-temperature photoluminescence (PL) spectra in the visible emission region were measured to investigate the oxygen vacancy-related and other defect states in the Ga_2_O_3_ nanorods. Figure 4 presents the PL spectra of the Ga_2_O_3_ nanorods grown on the planar and various roughened SnO_2_ seed layers. In general, the luminescence observed in the visible region has been attributed to defect-related radiative recombination involving oxygen vacancy-related defects and other intrinsic defects in Ga_2_O_3_ [32,33]. As shown in Figure 4, Ga_2_O_3_ nanorods grown on the 800 nm periodic SnO_2_ seed layer exhibit the strongest visible luminescence intensity, followed by those on the 600 and 1000 nm periodic SnO_2_ seed layers, while the nanorods grown on the planar SnO_2_ seed layer show the weakest visible emission. Since the visible luminescence intensity was positively correlated with the number of oxygen vacancy-related defects, these experimental results indicated that the Ga_2_O_3_ nanorods grown on an 800 nm periodic SnO_2_ seed layer had the largest total number of oxygen vacancy-related defects, followed by those grown on the 600 and 1000 nm periodic SnO_2_ seed layers, whereas Ga_2_O_3_ nanorods grown on the planar SnO_2_ seed layer possessed the smallest total number of oxygen vacancy-related defects. Since Ga_2_O_3_ nanorods were grown on the various SnO_2_ seed layers using the same hydrothermal synthesis process, it could be assumed that there was a similar local surface chemical-state fraction of a Ga_2_O_3_ nanorod per unit area. Therefore, compared with the planar SnO_2_ seed layer, it could be inferred that more Ga_2_O_3_ nanorods were grown on the roughened SnO_2_ seed layers. Furthermore, different periodic SnO_2_ seed layers could also grow different numbers of Ga_2_O_3_ nanorods.

**Figure 5:**
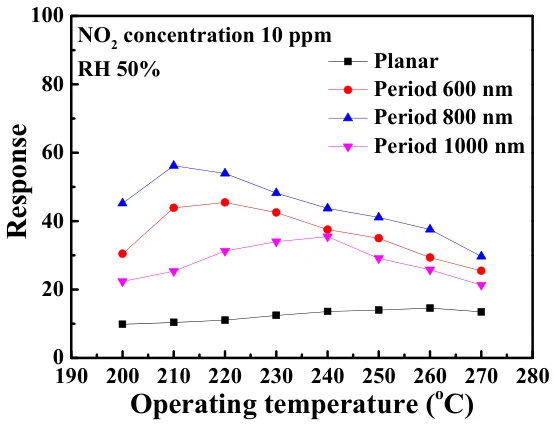
Response as a function of operating temperature of NO_2_ gas sensors fabricated using Ga_2_O_3_ nanorods grown on planar and roughened SnO_2_ seed layers with different periods.

The response of NO_2_ gas sensors is expressed as:

Response = R_g_/R_a_ (10)

where R_g_ and R_a_ are the resistances of the sensors in the NO_2_ and air environments, respectively. At a NO_2_ concentration of 10 ppm and a relative humidity (RH) of 50%, Figure 5 shows the response as a function of operating temperature for the resulting NO_2_ gas sensors operating at a bias voltage of 1 V. The responses of the NO_2_ gas sensors fabricated using Ga_2_O_3_ nanorods grown on planar, 600, 800, and 1000 nm periodic SnO_2_ seed layers were 14.6, 45.5, 56.2, and 35.5, respectively. The response trend was consistent with the trend in the number of oxygen vacancy-related defects induced by Ga_2_O_3_ nanorods grown on various SnO_2_ seed layers. Since nanorods with a larger total surface provided more adsorption sites of oxygen vacancy-related defects [34,35,36], both the results of the morphological and photoluminescence analyses indicated that the Ga_2_O_3_ nanorods grown on the 800 nm periodic SnO_2_ seed layer possessed the largest content of oxygen vacancy-related defects. Consequently, these Ga_2_O_3_ nanorods provided the most gas adsorption sites and exhibited the highest response. In comparison, the Ga_2_O_3_ nanorods grown on the 600 and 1000 nm periodic SnO_2_ seed layers contained fewer adsorption sites than those on an 800 nm periodic SnO_2_ seed layer, resulting in relatively lower enhancements in NO_2_ gas response.

**Figure 6:**
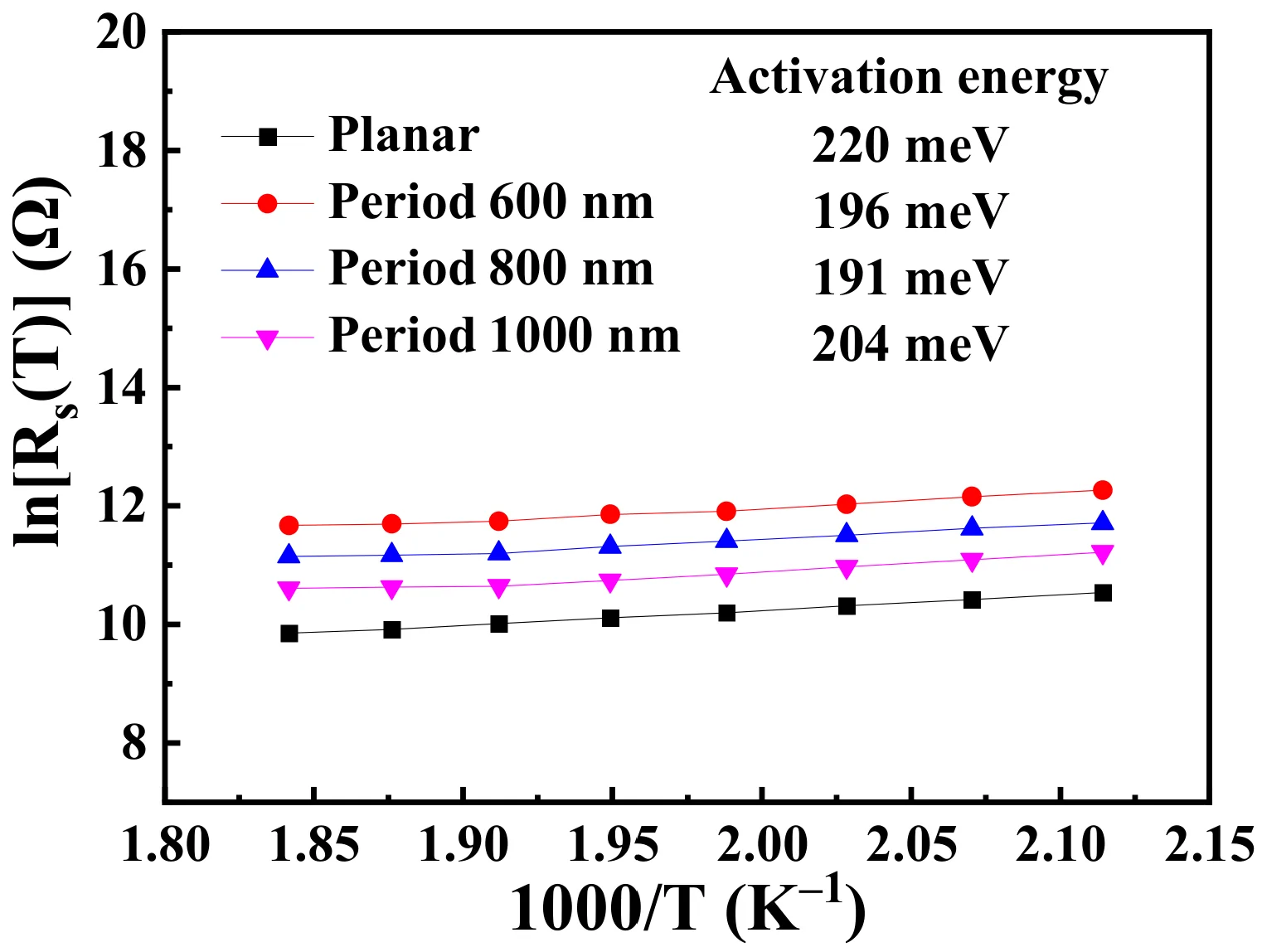
Temperature-dependent resistance of NO_2_ gas sensors fabricated using Ga_2_O_3_ nanorods grown on planar and roughened SnO_2_ seed layers with different periods.

**Figure 7:**
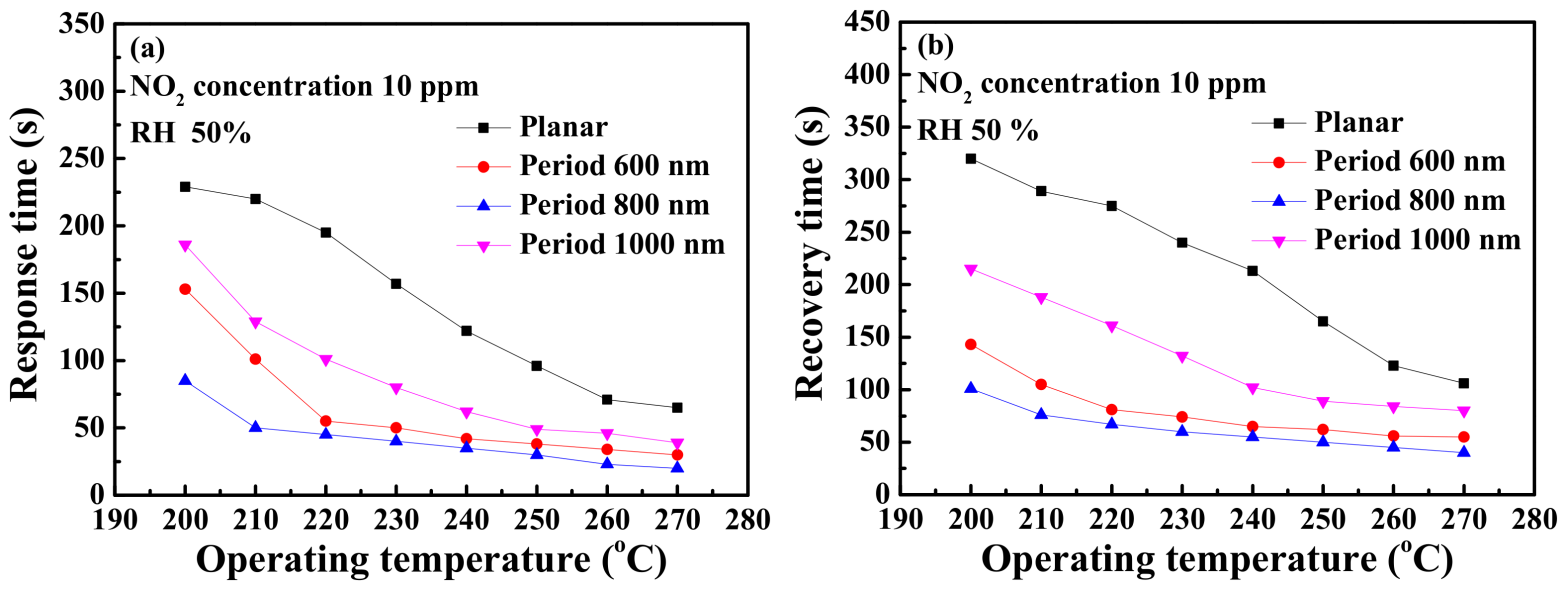
(**a**) Response time and (**b**) recovery time of NO_2_ gas sensors fabricated using Ga_2_O_3_ nanorods grown on planar and roughened SnO_2_ seed layers with different periods.

Figure 6 illustrates the temperature-dependent resistance (R_s_(T)) of the resulting various NO_2_ gas sensors. Generally, both the reaction rate and the operating temperature were predominantly influenced by the activation energy (E_A_). The activation energy was calculated from the slope of the Arrhenius plot expressed as [37,38]:

(11) $$R_{s}(T)\;=\;R_{0}\;\exp(\frac{E_{A}}{\mathrm{kT}})$$

(12) $$\ln[R_{s}(T)]=\ln(R_{0})+(\frac{E_{A}}{1000k})\;(\frac{1000}{T})$$

where R_0_ is the pre-exponential factor, k is the Boltzmann constant, and T is the absolute temperature. As shown in Figure 6, the activation energy of the NO_2_ gas sensors fabricated using the Ga_2_O_3_ nanorods grown on planar, 600, 800, and 1000 nm periodic SnO_2_ seed layers was 220, 196, 191, and 204 meV, respectively. It was found that the decreasing activation energy trend followed the increasing trend of the number of Ga_2_O_3_ nanorods and oxygen vacancy-related defects. This could be attributed to a reduction in the reaction energy barrier due to the promoted formation of more oxygen vacancy-related defects and additional homojunctions between the Ga_2_O_3_ nanorods. As shown in Figure 5, the individual optimal operating temperature of the NO_2_ gas sensor fabricated using the Ga_2_O_3_ nanorods grown on planar, 600, 800, and 1000 nm periodic SnO_2_ seed layers was 260 °C, 220 °C, 210 °C, and 240 °C, respectively. The decreasing trend in the optimal operating temperature was consistent with the reduction trend of the associated activation energy. Furthermore, the lower activation energy indicated that less energy was required to initiate the surface reaction. Figure 7 presents the response time and recovery time of the various NO_2_ gas sensors. The response time was defined as the required time for the response value to change to 90% of the maximum response value, while the recovery time was defined as the required time for the response value to change from 100% to 10% of the maximum response value. At their respective optimal operating temperatures of 260 °C, 220 °C, 210 °C, and 240 °C, the response times were measured to be 71, 55, 50, and 62 s, while the recovery times were 123, 81, 76, and 102 s for the planar, 600, 800, and 1000 nm periodic SnO_2_ seed layers, respectively. Both the response and recovery times were shortened by the reduction in the activation energy. This improvement in switching speed could be attributed to their lower activation energies, which facilitated faster adsorption and desorption processes of NO_2_ molecules, thereby reducing both the response and recovery times.

**Figure 8:**
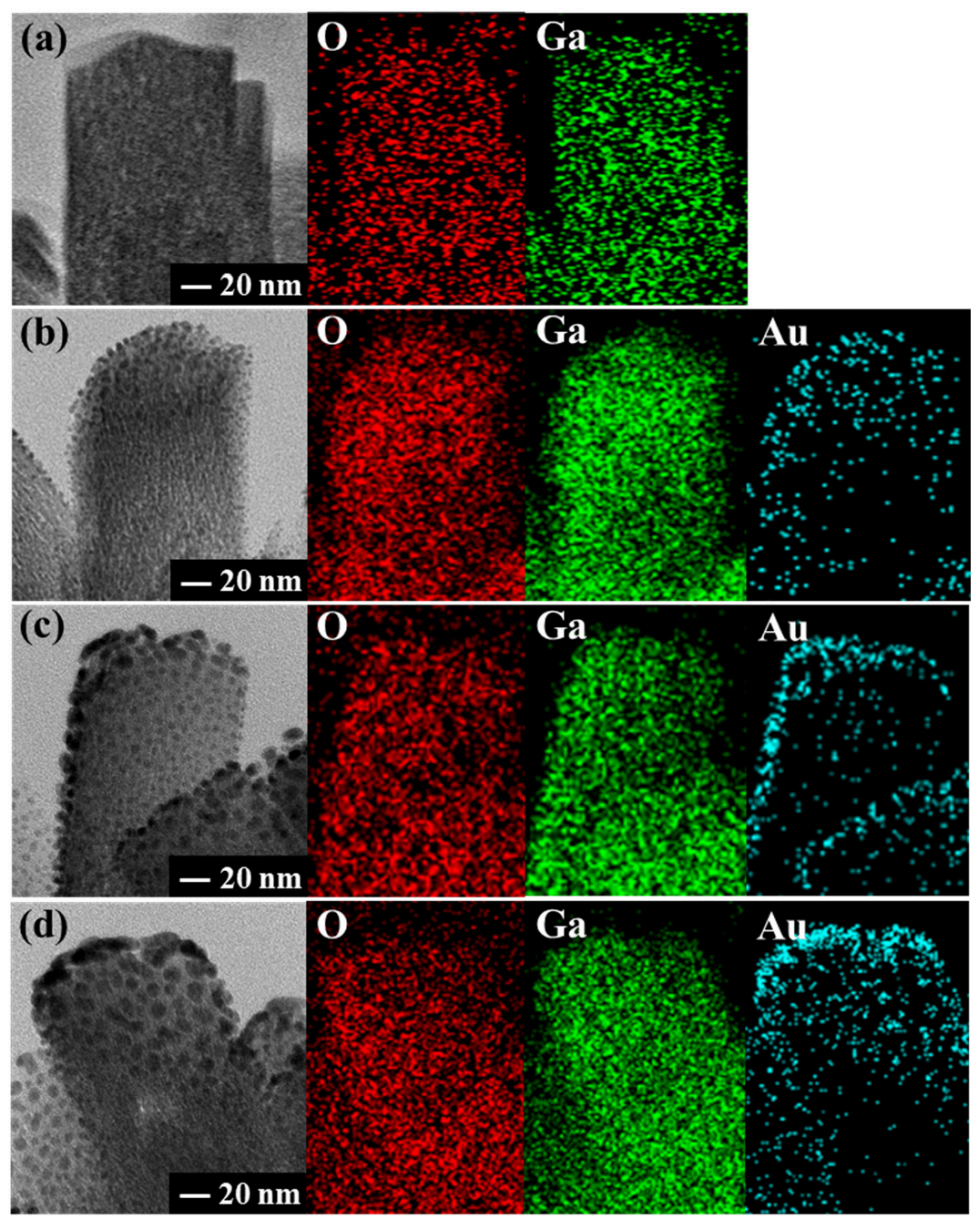
HRTEM cross-sectional images and two-dimensional elemental mapping images of gold-black nanoparticles deposited on Ga_2_O_3_ nanorods for (**a**) 0 s, (**b**) 30 s, (**c**) 60 s, and (**d**) 90 s.

To study the spillover effect and heterojunction effect, p-type gold-black NPs were deposited on the n-type Ga_2_O_3_ nanorods grown on the 800 nm periodic SnO_2_ seed layer for various times using a vapor cooling condensation system. Figure 8a–d illustrate the HRTEM cross-sectional images and the two-dimensional elemental mapping images of the Ga_2_O_3_ nanorods decorated with gold-black NPs deposited for 0, 30, 60, and 90 s, respectively. Both the surface coverage and the particle size of the gold-black NPs increased with longer deposition times. Table 1 presents the atomic percentages of gallium (Ga), oxygen (O), and Au on the Ga_2_O_3_ nanorods decorated with gold-black NPs for various times, as determined by the EDS analysis. When the deposition times were 30, 60, and 90 s, the Au contents were 0.68, 1.02, and 1.39 at.%, respectively. The results indicated that the gold-black NPs content increased with a longer deposition time.

**Figure 9:**
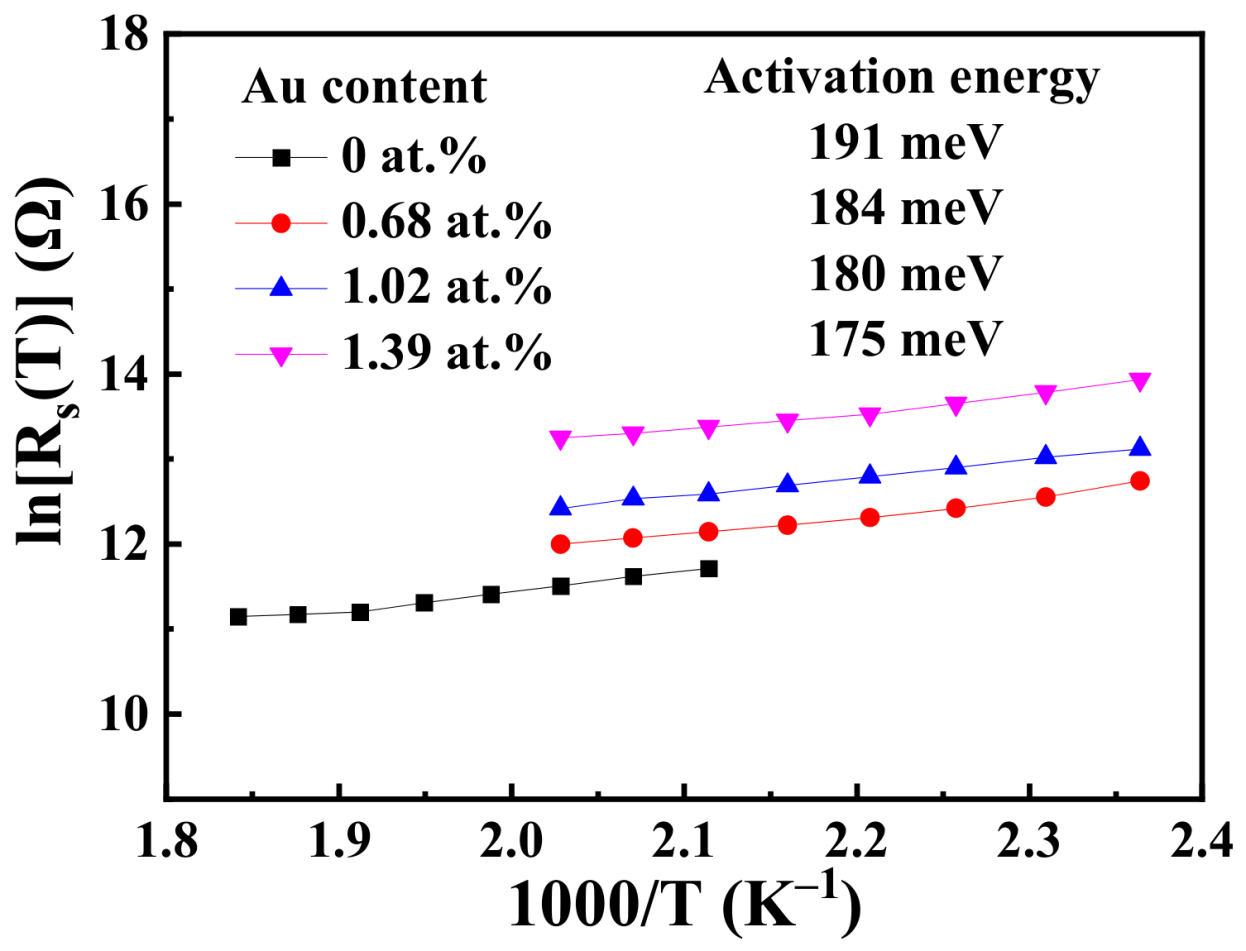
Temperature-dependent resistance of NO_2_ gas sensors fabricated using Ga_2_O_3_ nanorods decorated with various gold-black nanoparticle contents.

**Figure 10:**
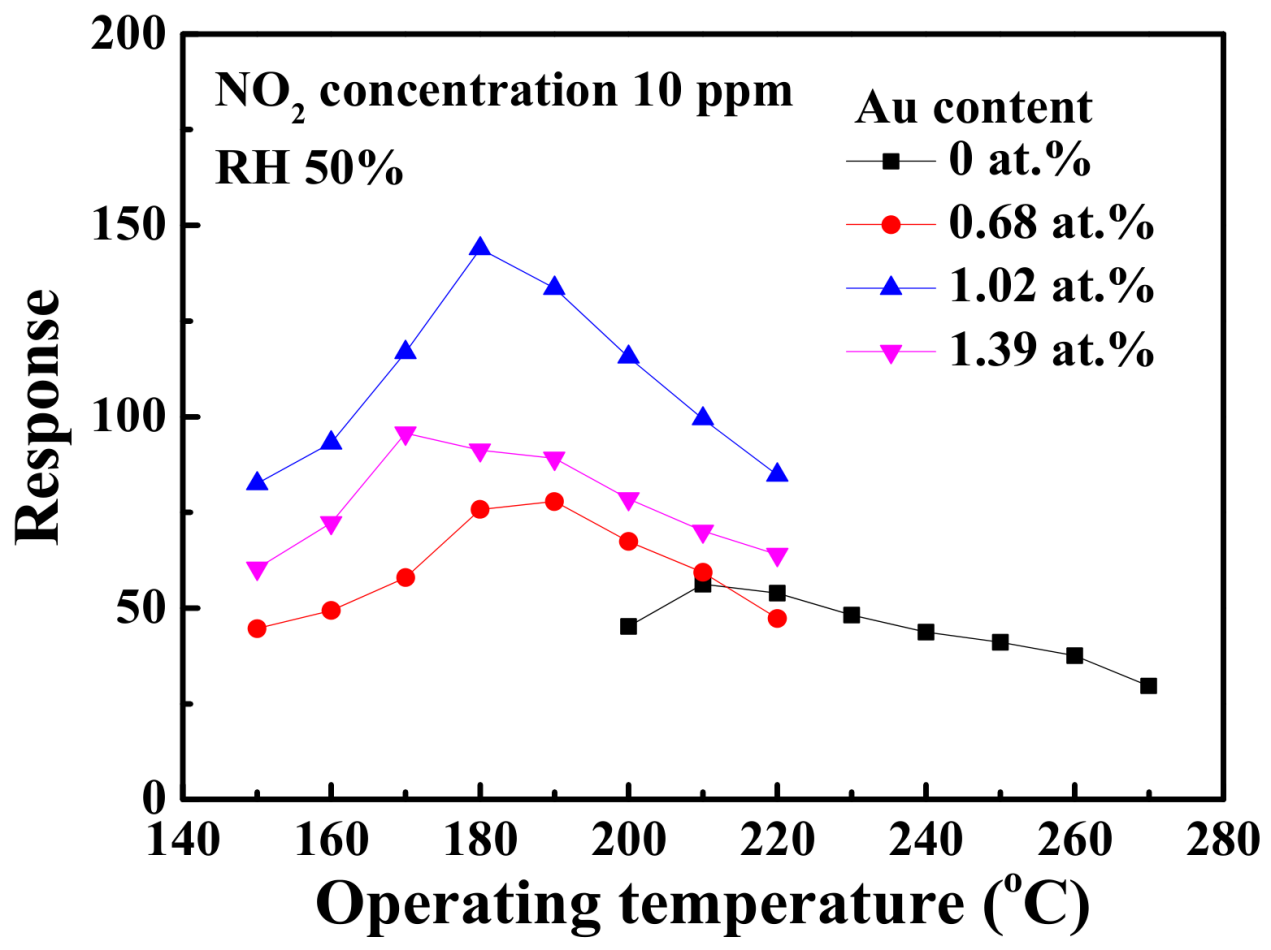
Response of NO_2_ gas sensors fabricated using Ga_2_O_3_ nanorods with various gold-black nanoparticle contents.

**Figure 11:**
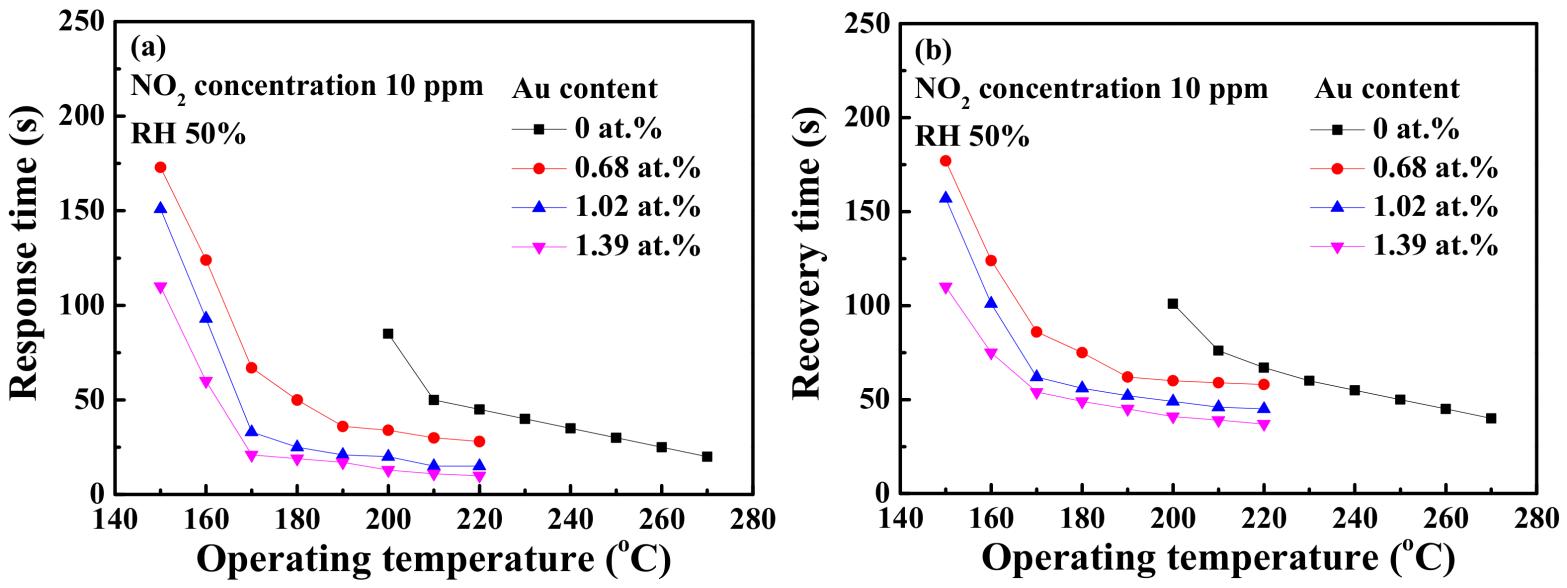
(**a**) Response time and (**b**) recovery time of NO_2_ gas sensors fabricated using Ga_2_O_3_ nanorods decorated with various contents of gold-black nanoparticles.

Figure 9 illustrates the temperature-dependent resistance R_s_(T) for the NO_2_ gas sensors fabricated using the Ga_2_O_3_ nanorods decorated with Au contents of 0, 0.68, 1.02, and 1.39 at.%. The corresponding activation energies were 191, 184, 180, and 175 meV. It was observed that the resistance of the NO_2_ gas sensors increased with increasing Au content. This experimental result verified that extra depletion regions were induced by the formation of additional p-n heterojunctions by decorating more p-type gold-black NPs on the n-type Ga_2_O_3_ nanorods, which in turn increased the resistance of the NO_2_ gas sensors. Additionally, it is noteworthy that the activation energy exhibited a decreasing trend as the number of gold-black NPs increased. This reduction in activation energy was attributed to the enhanced spillover effect, as noble gold-black NPs are known to possess excellent catalytic properties [39,40,41]. At a NO_2_ concentration of 10 ppm and an RH of 50%, Figure 10 shows the response as a function of operating temperature for various NO_2_ gas sensors operated at a bias voltage of 1 V. The responses of the NO_2_ gas sensor fabricated using Ga_2_O_3_ nanorods decorated with Au content of 0, 0.68, 1.02, and 1.39 at.% were 56.2, 77.9, 143.9, and 95.7, respectively. The corresponding optimal operating temperatures were 210, 190, 180, and 170 °C, respectively. The improvement in response could be attributed to the spillover effect and catalytic effect of the gold-black NPs. When oxygen molecules were adsorbed on the gold-black NPs, they could be catalyzed into adsorbed oxygen ions $O_{2(\mathrm{ads})}^{-}$ and $O_{\left(\mathrm{ads}\right)}^{-}$ that were diffused to the Ga_2_O_3_ nanorod surface to work as additional adsorption sites for NO_2_ molecules. This process could accelerate the thermodynamical adsorption of ${\mathrm{NO}}_{2(\mathrm{ads})}^{-}$ as indicated in Equation (9). Therefore, due to the assistant effect of gold-black NPs in sensing processes and the formation of a larger number of p-n heterojunctions in the gold-black NPs decorated Ga_2_O_3_ nanorods, the response increased with an increase in Au content to 1.02 at.%. However, when the Au content further increased from 1.02 at.% to 1.39 at.%, the response decreased from 143.9 to 95.7. This phenomenon was attributed to excessive gold-black NPs covering the gas adsorption sites on the surface of the Ga_2_O_3_ nanorods, thereby decreasing the response of NO_2_ gas sensors. Moreover, the optimal operating temperature decreased from 210 °C to 170 °C as the Au content increased from 0 to 1.39 at.%. Gold-black NPs acted catalytically; more gold-black NPs could catalyze and activate more adsorbed oxygen molecules into chemisorbed oxygen ions, which could diffuse to the surface of the Ga_2_O_3_ nanorods. The associated activation energy for the surface reaction between NO_2_ and Ga_2_O_3_ lattice was reduced owing to the improvement of the NO_2_ gas reaction. In general, the lower activation energy indicated that charge carriers required less energy to overcome the energy barrier, allowing the sensing mechanisms to operate at a lower temperature [42]. Figure 11a,b present the response time and recovery time of the NO_2_ gas sensors fabricated using the Ga_2_O_3_ nanorods decorated with various Au contents, respectively. At their respective optimal operating temperatures of 210, 190, 180, and 170 °C, the response times were measured as 50, 36, 25, and 21 s, while the recovery times were 76, 62, 56, and 54 s for the Au content of 0, 0.68, 1.02, and 1.39 at.%, respectively. These results revealed that incorporating gold-black NPs effectively reduced both the response and recovery times of the resulting NO_2_ gas sensors. As the deposition time of the gold-black NPs increased, the amount and size of the gold-black NPs also increased, thereby reducing the activation energy and subsequently accelerating the sensing reaction behavior of NO_2_ gas sensors.

**Figure 12:**
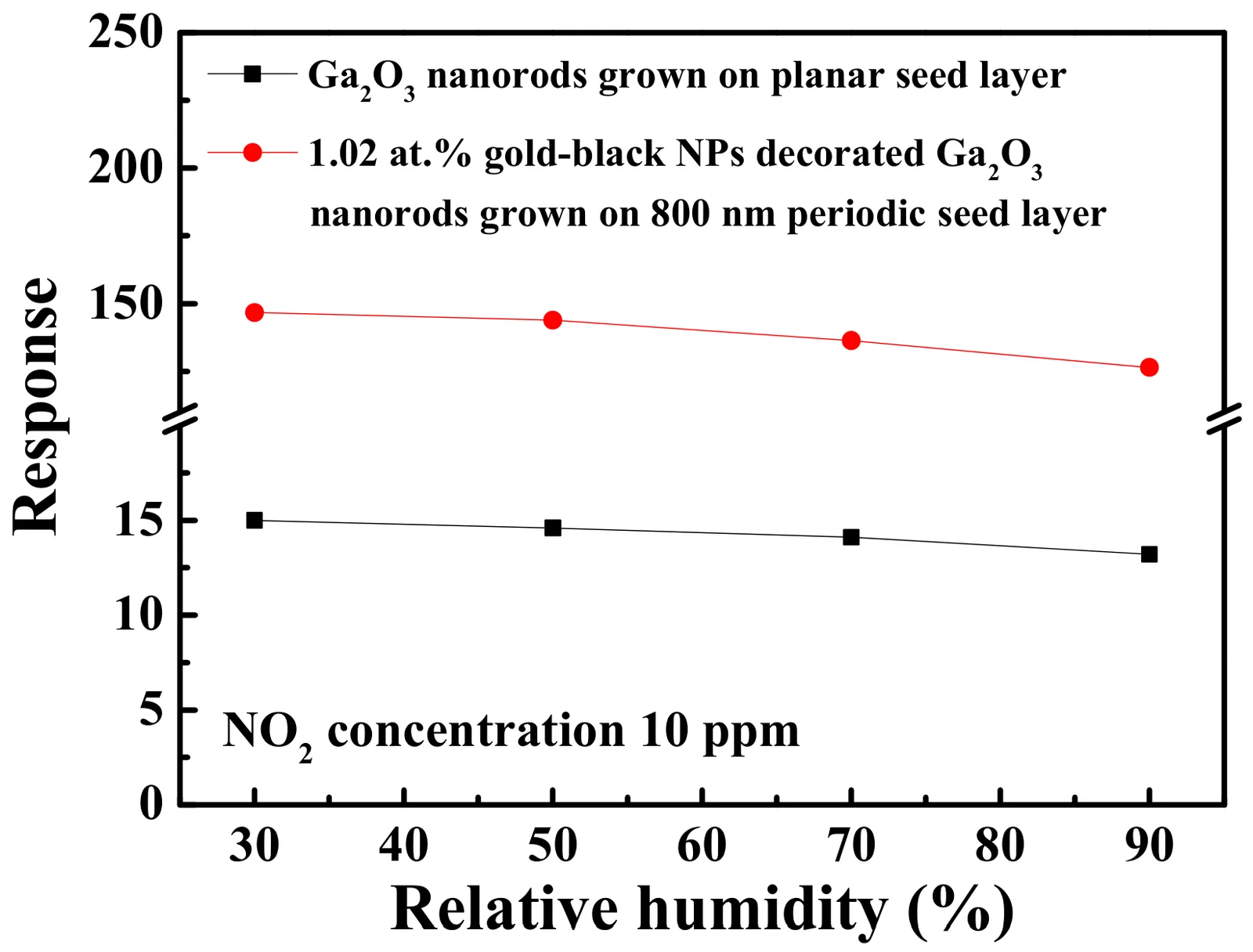
RH-dependent response of NO_2_ gas sensors fabricated using Ga_2_O_3_ nanorods grown on planar SnO_2_ seed layer and Ga_2_O_3_ nanorods grown on 800 nm periodic SnO_2_ seed layer decorated with 1.02 at.% Au content.

Stability is one of the critical factors that determines whether a gas sensor can be applied in practical environments. This study investigated the stability of the effect of relative humidity on the gas sensor performance. Figure 12 shows the RH-dependent response of the NO_2_ gas sensors fabricated using the Ga_2_O_3_ nanorods grown on the planar SnO_2_ seed layer operating at 260 °C and the Ga_2_O_3_ nanorods grown on the 800 nm periodic SnO_2_ seed layer decorated with 1.02 at.% Au content operating at 180 °C. The results revealed that as RH increased, the response of the both NO_2_ gas sensors declined. For the NO_2_ gas sensors fabricated using the Ga_2_O_3_ nanorods grown on the planar SnO_2_ seed layer, the response decreased from 15.0 to 13.2 as RH increased from 30% to 90%, a 12.0% decline. Similarly, for the NO_2_ gas sensors fabricated with gold-black NPs/Ga_2_O_3_ nanorods, the response decreased from 146.7 to 126.5 when RH increased from 30% to 90%, a 13.8% decline. This experimental result was attributed to water-molecule adsorption and dissociation on the Ga_2_O_3_ nanorod surfaces, in which the resulting OH^−^ ions replaced chemisorbed oxygen species, releasing electrons back into the conduction band of the Ga_2_O_3_ nanorods and occupying active reaction sites, thereby reducing the sensing response [43,44].

**Figure 13:**
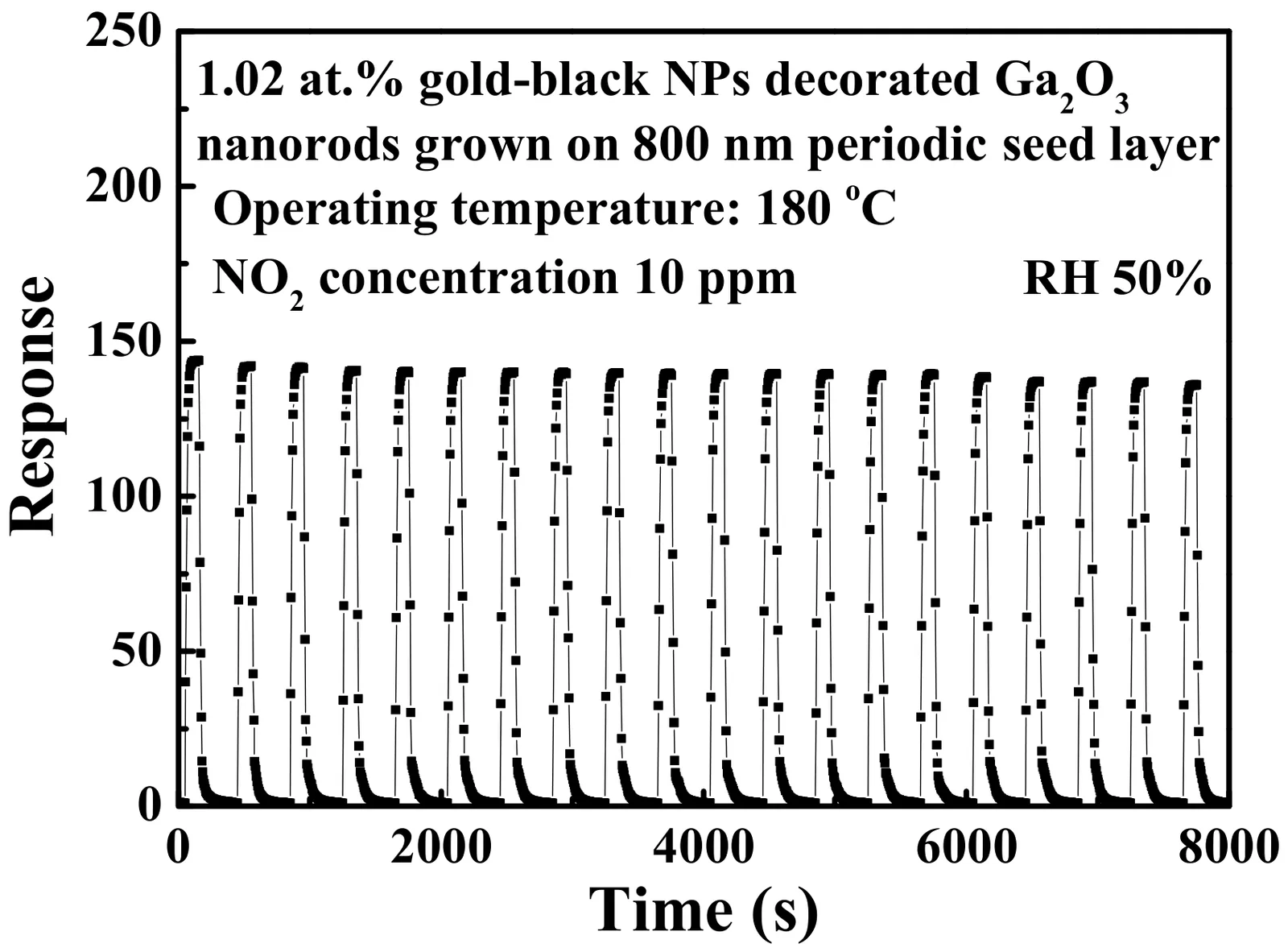
Response of NO_2_ gas sensor fabricated using 1.02 at.% gold-black NPs/Ga_2_O_3_ nanorods after 20 cycles over 2 h.

To further verify the operational stability of the NO_2_ gas sensors, short-term repeatability tests were conducted on sensors fabricated using Ga_2_O_3_ nanorods grown on an 800 nm periodic SnO_2_ seed layer decorated with 1.02 at.% Au content. Under a NO_2_ concentration of 10 ppm, an RH of 50%, and an operating temperature of 180 °C, Figure 13 shows the response of the NO_2_ gas sensor tested continuously over 2 h across 20 measurement cycles. As shown in Figure 13, the response remained stable, with values of 143.9, 139.8, and 136.0 for the 1st, 10th, and 20th cycles, respectively, and only a 5.5% decrease from the initial response to the final response. These results demonstrated that the NO_2_ gas sensor had excellent short-term stability and repeatability.

**Figure 14:**
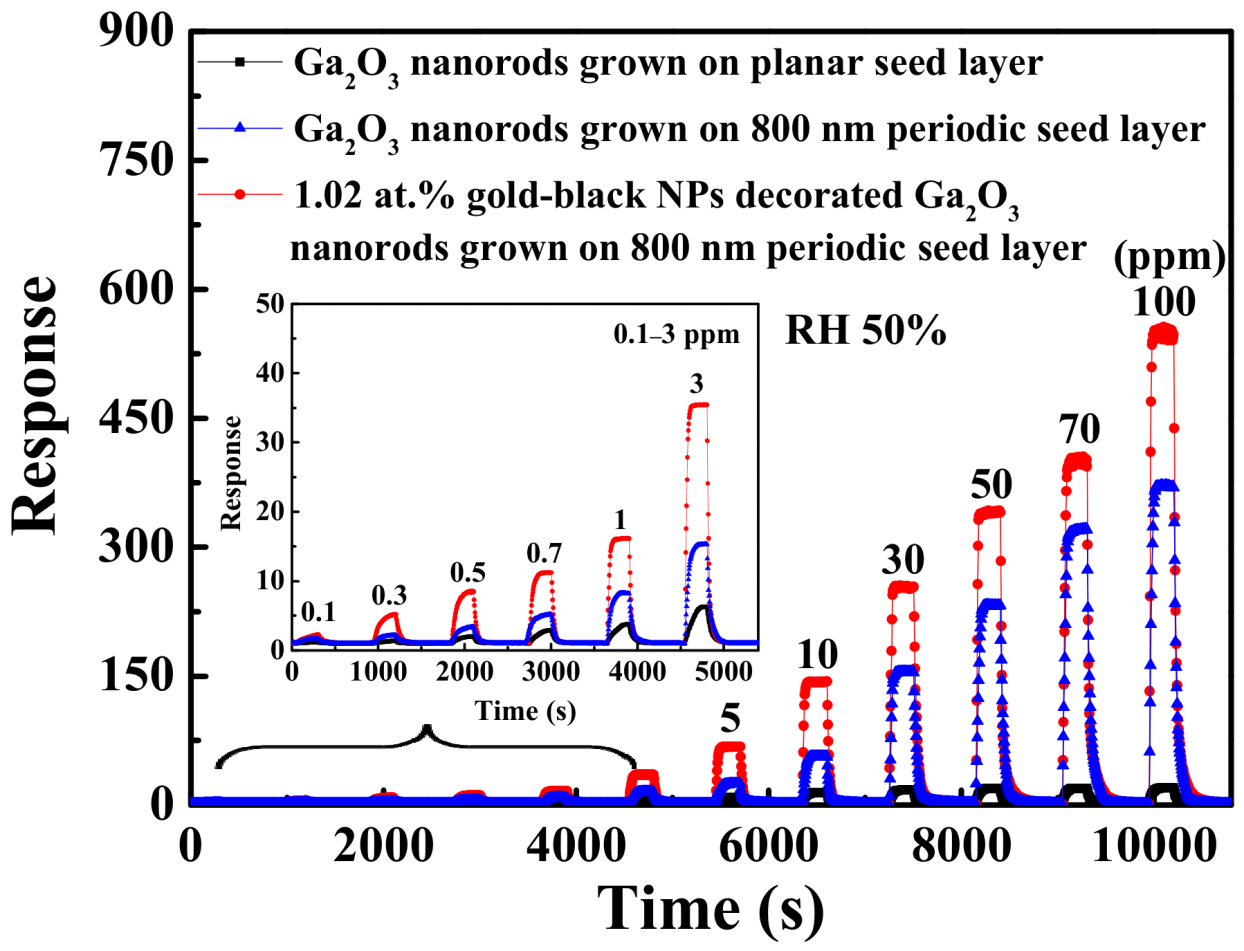
Dynamic response of NO_2_ gas sensor in different NO_2_ concentrations. Inset shows the extended response at very low NO_2_ concentrations.

Figure 14 shows the dynamic response of the three NO_2_ gas sensors fabricated using the Ga_2_O_3_ nanorods separately grown on the planar SnO_2_ seed layer and the 800 nm periodic SnO_2_ seed layer without and with decoration of 1.02 at.% Au content, operating at individual optimal temperatures of 260, 210, and 180 °C. As the NO_2_ concentration increased, all three NO_2_ gas sensors exhibited an increasing response trend. However, the NO_2_ gas sensor fabricated with Ga_2_O_3_ nanorods grown on the planar SnO_2_ seed layer exhibited a saturated response of 19.0 at concentrations above 50 ppm, indicating a limited sensing range. In contrast, the other two NO_2_ gas sensors continued to respond at higher NO_2_ concentrations, attributed to the increased number of available adsorption sites provided by the enhanced surface morphology and decoration of gold-black NPs. Notably, the NO_2_ gas sensor with gold-black NPs/Ga_2_O_3_ nanorods consistently exhibited the highest response across all NO_2_ concentrations, suggesting superior performance for detecting both high and low NO_2_ concentrations. At the 100 ppm NO_2_ concentration, the response of the NO_2_ gas sensors was increased from 372.2 to 556.2 by decorating 1.02 at.% Au content on the Ga_2_O_3_ nanorods grown on the 800 nm periodic SnO_2_ seed layer. The inset of Figure 14 illustrates the extended response of the three NO_2_ gas sensors at very low NO_2_ concentrations. At the lowest NO_2_ concentration of 100 ppb, the corresponding responses were 1.1, 1.7, and 2.3.

**Figure 15:**
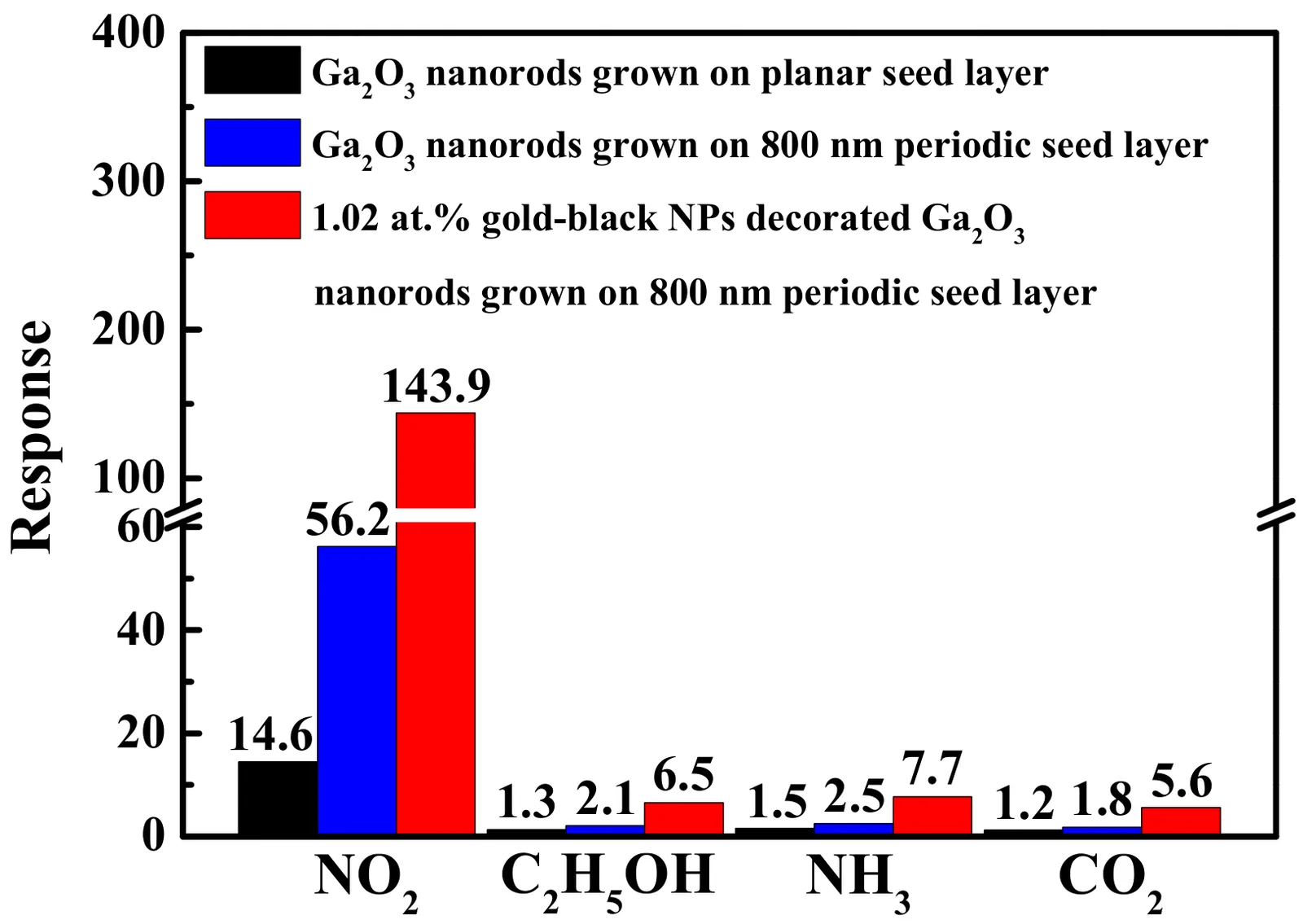
Response of various NO_2_ gas sensors under various target gases.

Figure 15 compares the gas selectivity of the two NO_2_ gas sensors fabricated using the Ga_2_O_3_ nanorods grown on the planar SnO_2_ seed layer operating at 260 °C, the Ga_2_O_3_ nanorods grown on the 800 nm periodic SnO_2_ seed layer operating at 210 °C, and the 1.02 at.% gold-black NPs/Ga_2_O_3_ nanorods grown on the 800 nm periodic SnO_2_ seed layer operating at 180 °C. Four different gases (10 ppm each) were introduced: two oxidizing gases (NO_2_ and carbon dioxide (CO_2_)) and two reducing gases (ethanol (C_2_H_5_OH) and ammonia (NH_3_)). Both gas sensors demonstrated high selectivity for NO_2_ gas. This could be explained by the differences in the bond dissociation energy of the target gases. According to the literature [45], NO_2_ gas has the lowest bond dissociation energy among the four gases, making it more reactive with surface oxygen species under the same thermal conditions. Although CO_2_ is also an oxidizing gas, its response was lower than that of the two reducing gases due to its high bond dissociation energy and thermodynamic stability [46], which hindered its interaction with the sensing layer in the absence of catalysts or high temperatures. Due to the spillover effect of the noble gold-black NPs, the associated response of the four tested gases was also enhanced.

**Figure 16:**
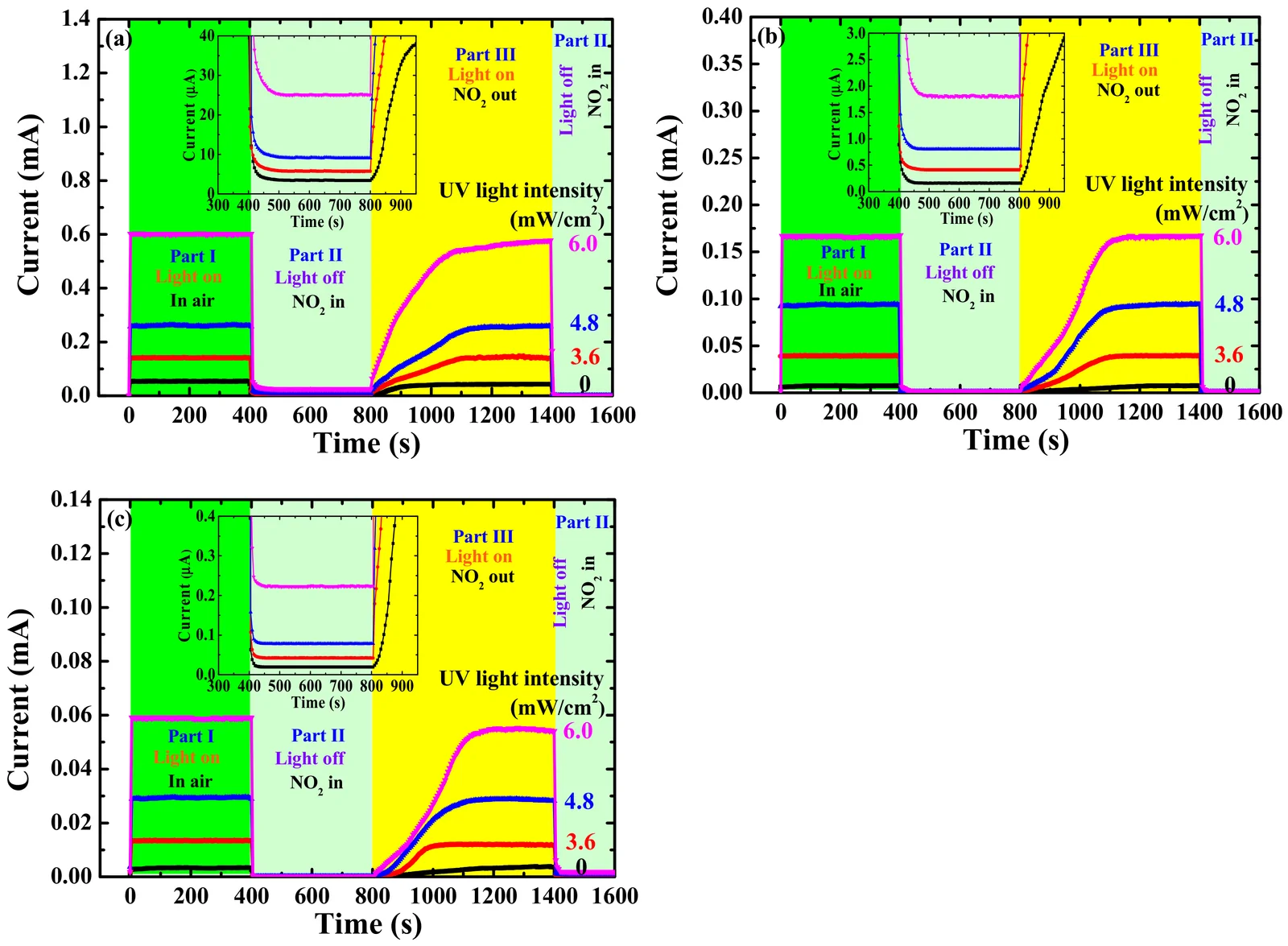
Response of NO_2_ gas sensors fabricated using Ga_2_O_3_ nanorods grown on (**a**) planar SnO_2_ seed layer and 800 nm periodic SnO_2_ seed layer (**b**) without and (**c**) with 1.02 at.% gold-black NPs decoration under dark and various UV light intensities.

**Figure 17:**
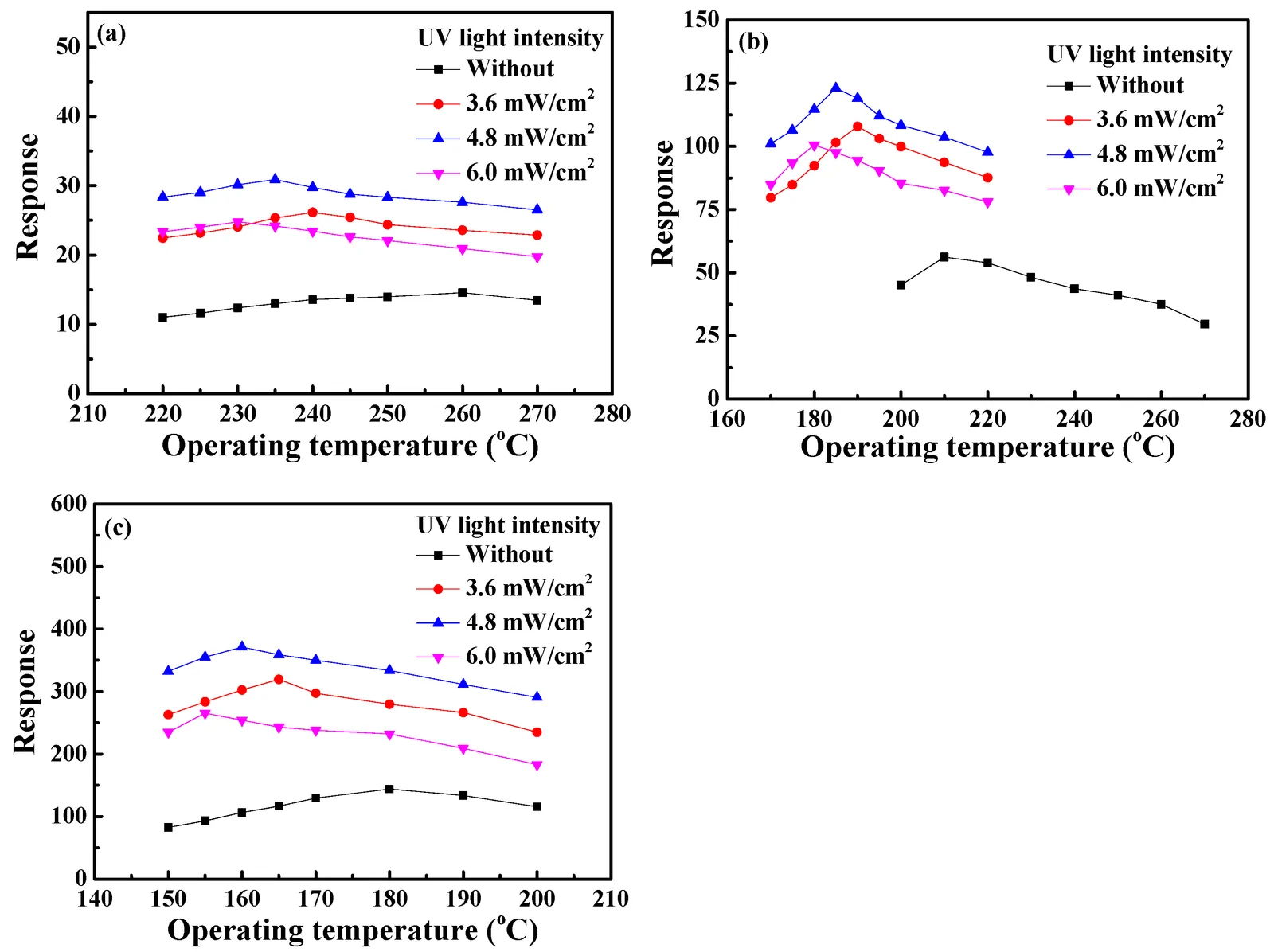
Under NO_2_ concentration of 10 ppm, RH of 50%, and bias voltage of 1 V, time-dependent current of NO_2_ gas sensors fabricated using (**a**) planar SnO_2_ seed layer and 800 nm periodic SnO_2_ seed layer (**b**) without and (**c**) with 1.02 at.% gold-black NPs decoration under dark and various UV light intensities.

The response of the NO_2_ gas sensors was defined as the ratio of the resistance R_g_ in the NO_2_ environment and the resistance R_a_ in the air environment. To enhance the response, reducing the NO_2_ gas sensor’s R_a_ and increasing its R_g_ were effective methods. Based on this method, we explored the characteristics and physical mechanisms of the enhanced effect of the photo-activated NO_2_ gas sensors. To reduce the R_a_ value, an intermittent ultraviolet (UV) light source (wavelength = 254 nm) with photon energy exceeding the Ga_2_O_3_ nanorod bandgap energy of 4.98 eV [47] was used in air before NO_2_ gas was introduced. To investigate the light-activated effect on response, the current–time (I–t) characteristics of the various NO_2_ gas sensors illuminated by the intermittent UV light intensities of 3.6, 4.8, and 6.0 mW/cm^2^ were measured under the NO_2_ concentration of 10 ppm, RH of 50%, and bias voltage of 1 V. Figure 16a–c show the associated I–t characteristics of the NO_2_ gas sensors fabricated using the Ga_2_O_3_ grown on the planar SnO_2_ seed layer and the 800 nm periodic SnO_2_ seed layer without and with decoration of 1.02 at.% Au content, respectively, under dark and various UV light intensities. In the light-activated I–t measurement, these various NO_2_ gas sensors were operated at their respective optimal operating temperatures, as listed in Table 2. The I–t measurement included three parts: Part I was the UV light turned on, and the chamber was in an air environment; Part II was the UV light turned off, and NO_2_ gas was introduced into the chamber; Part III was the UV light turned on, and the NO_2_ gas was removed. In part I, the electron-hole pairs generated by UV light not only increased the conductivity of the Ga_2_O_3_ nanorod by raising the carrier concentration but also reduced the Debye depletion length, thereby increasing the current and decreasing the resistance R_a_ of the NO_2_ gas sensors. Furthermore, because higher UV light intensity could generate more electron-hole pairs, the current increased with UV light intensity. Eventually, they all reached a saturated current value in equilibrium. The associated resistance R_a_ for the experimental results shown in Figure 16 was listed in Table 2. It was found that the R_a_ of all the NO_2_ gas sensors decreased just like the current increased as the UV light intensity increased. In part II, after the UV light was turned off and NO_2_ gas was introduced to the NO_2_ gas sensors, the current of all sensors decreased rapidly. It was attributed to the rapid recombination of electrons and holes generated by UV light, and to the introduced NO_2_ molecules, which captured electrons from the conduction band of the Ga_2_O_3_ nanorods, as shown in Equation (8), thereby further reducing the conductivity of the Ga_2_O_3_ nanorods. In addition, NO_2_ molecules also reacted with adsorbed oxygen ions $O_{\left(\mathrm{ads}\right)}^{-}$ and formed O_2_ gas, as shown in Equation (9). According to Equation (5), the O_2_ molecules could retransform to the adsorbed O_2_, which would react with electrons to form the $O_{\left(\mathrm{ads}\right)}^{-}$, as shown in Equations (6) and (7). Consequently, the resulting current decreased, and the associated resistance R_g_ of the NO_2_ gas sensors increased. The related resistance R_g_ is listed in Table 2. The inset of Figure 16a–c show the associated current of the various NO_2_ gas sensors after the UV light was turned off and the NO_2_ gas was introduced. As shown in the inset of Figure 16a–c, after UV light was turned off and NO_2_ gas was introduced, the currents of all three kinds of NO_2_ gas sensors under various UV light intensities were larger than those without UV light illumination. In addition, the current was increased with increasing UV light intensity. This phenomenon was attributed to the presence of persistent photocurrent (PPC) [48]. Subsequently, in part III, when the UV light was turned on and the NO_2_ gas was removed, the current of the NO_2_ gas sensors under UV light illumination increased rapidly compared to that of the NO_2_ gas sensors without UV light illumination. Because the photogenerated holes caused by the UV light illumination would facilitate the desorption of preadsorbed $O_{\left(\mathrm{ads}\right)}^{-}$ and ${\mathrm{NO}}_{2(\mathrm{ads})}^{-}$ ions [25,49], the sensing surface of Ga_2_O_3_ nanorods was refreshed. Therefore, the UV light assistance could reduce the recovery time of the gas sensors. From the experimental results for R_a_ and R_g_, Figure 17a–c present the response as a function of operating temperature for the various NO_2_ gas sensors under dark and illuminated conditions at various UV light intensities. The maximum response and the optimal operating temperature were also listed in Table 2. It was worth noting that the response was enhanced and affected by illuminating UV light on the NO_2_ gas sensors. The enhanced response reached its maximum at the UV intensity of 4.8 mW/cm^2^, then decreased as the UV intensity increased further to 6.0 mW/cm^2^. This phenomenon was attributed to the resistance R_g_ of all NO_2_ gas sensors illuminated at a UV intensity of 6.0 mW/cm^2^ being lower than that of those illuminated at 4.8 mW/cm^2^. The optimal operating temperature was reduced by illuminating the various NO_2_ gas sensors with higher UV light intensity. This phenomenon was attributed to the photogenerated electrons and holes participating in surface-sensing reactions, thereby reducing the activation energy required for gas sensing. Consequently, as higher UV light intensity produced more electron-hole pairs, the activation energy and the corresponding optimal operating temperature decreased.

## 4. Conclusions

In summary, we studied and enhanced the performance of NO_2_ gas sensors fabricated using Ga_2_O_3_ nanorods grown onto nanoimprinted SnO_2_ seed layers with various periods. Compared with the response of 14.6 for the NO_2_ gas sensors using the Ga_2_O_3_ nanorods grown on the planar SnO_2_ seed layers, the response was enhanced to 56.2 for the NO_2_ gas sensors using Ga_2_O_3_ nanorods grown on an 800 nm periodic SnO_2_ seed layer. The improvement in response was attributed to the growth of more tilted Ga_2_O_3_ nanorods on the sidewalls of the periodic SnO_2_ seed layer and the formation of more homojunction connectors. To promote electron transfer and catalyze surface reactions by incorporating gold-black NPs on the Ga_2_O_3_ nanorod surface, the response was further enhanced to 143.9 for the NO_2_ gas sensors fabricated by 1.02 at.% gold-black NPs/Ga_2_O_3_ nanorods grown on the 800 nm periodic SnO_2_ seed layer. To further improve the response of NO_2_ gas sensors, increasing the carrier concentration to reduce the resistance R_a_ in air was an effective method. To complement and apply this method, the resulting NO_2_ gas sensors were irradiated with UV light at a wavelength of 254 nm to generate electron-hole pairs, thereby increasing the concentration of conductive carriers in the air. Under the UV light intensity of 4.8 mW/cm^2^, the response was increased from 14.6 to 30.9 and from 56.2 to 123.1 for the NO_2_ gas sensors fabricated using Ga_2_O_3_ nanorods grown on a planar SnO_2_ seed layer and an 800 nm periodic SnO_2_ seed layer, respectively. Furthermore, the response was increased from 143.9 to 370.5 for the NO_2_ gas sensors fabricated using 1.02 at.% gold-black NPs/Ga_2_O_3_ nanorods grown on an 800 nm periodic SnO_2_ seed layer. Just like the mechanisms for improving response, the optimal operating temperature of the NO_2_ gas sensors was also improved. Our experimental results suggested that SnO_2_ seed layers with different periodic groove patterns could generate different numbers of Ga_2_O_3_ nanorods and homojunction connectors, thereby improving the response of the resulting NO_2_ gas sensors. However, to obtain the best response characteristics of the resulting NO_2_ gas sensors, theoretical simulation analysis of the period is still needed based on the length, diameter, and density of the Ga_2_O_3_ nanorods, as well as the tilt angle of the Ga_2_O_3_ nanorods growing on the sidewalls. Although irradiating the Ga_2_O_3_ nanorods used to fabricate NO_2_ gas sensors with UV light can improve sensing characteristics, simulation analysis of the optimal UV light intensity based on data such as the electron concentration, length, and diameter of the Ga_2_O_3_ nanorods is still necessary to achieve optimal sensing characteristics and sensing speed. Moreover, although quantitative experimental results were implemented in this study, further in-depth and detailed exploration is needed to obtain the overall optimal sensing characteristics. Even so, the results of this study, which employed various experimental conditions to conduct quantitative experiments, still serve as a foundation for leading improvements in gas sensor characteristics.

## Figures and Tables

**Table 1:** Gallium, oxygen, and gold atomic percentages of Ga_2_O_3_ nanorods decorated with gold-black NPs for various times.

Gold-Black NPs Deposition Time (s)	Ga (at.%)	O (at.%)	Au (at.%)
0	44.72	55.28	X
30	44.06	55.26	0.68
60	43.95	55.03	1.02
90	43.65	54.96	1.39

**Table 2:** Initial R_a_ and R_g_, optimal operating temperature, and maximum response in 10 ppm NO_2_ concentration under UV light illumination with various light intensities.

UV Light Intensity (mW/cm^2^)	Operating Temperature (°C)	R_a_ (kΩ)	R_g_ (kΩ)	Response R_g_/R_a_
Ga_2_O_3_ nanorods grown on a planar seed layer				
Dark	260	20.2	294.9	14.6
3.6	240	6.7	174.9	26.1
4.8	235	3.6	111.2	30.9
6.0	230	1.6	39.7	24.8
Ga_2_O_3_ nanorods grown on 800 nm periodic SnO_2_ seed layer				
Dark	210	111.2	6249.4	56.2
3.6	190	22.5	2423.3	107.7
4.8	185	10.1	1243.3	123.1
6.0	180	5.5	553.3	100.6
1.02 at.% gold-black NPs/Ga_2_O_3_ nanorods grown on 800 nm periodic SnO_2_ seed layer				
Dark	180	365.0	52,523.5	143.9
3.6	165	74.6	23,797.4	319.0
4.8	160	34.6	12,819.3	370.5
6.0	155	16.9	4498.8	266.2

## Data Availability

The data presented in this study are available upon request from the corresponding author.
